# Anaphylaxis from Wasp Stings Inducing Coronary Thrombus

**DOI:** 10.1155/2012/701753

**Published:** 2012-06-11

**Authors:** Sazzli Kasim, Rafidah AbuBakar, Eugene McFadden

**Affiliations:** ^1^Department of Cardiology, Universiti Teknologi MARA, Sg Buloh, Selangor, Malaysia; ^2^Department of Cardiology, Cork University Hospital, Cork, Ireland

## Abstract

Myocardial infarction as a result of wasp stings is a rare manifestation of acute coronary syndromes. It has been ascribed to kounis syndrome or allergic angina whose triggers include mast cell degranulation leading to coronary vasospasm and/or local plaque destabilisation. Its exact pathophysiology is still not clearly defined. We present a case of an acute coronary syndrome as a consequence of wasp stings and discuss its possible aetiology.

## 1. Introduction

Stenting with drug-eluting stents has almost become the default treatment of choice for acute coronary syndromes in patients eligible for PCI. Plaque instability, however, may not be the only underlying pathological process with a myriad of other factors involved in the haemostasis cascade. Considerations for the individual patient are paramount in delivering optimal treatment.

## 2. Case Report

**Figure 1:**
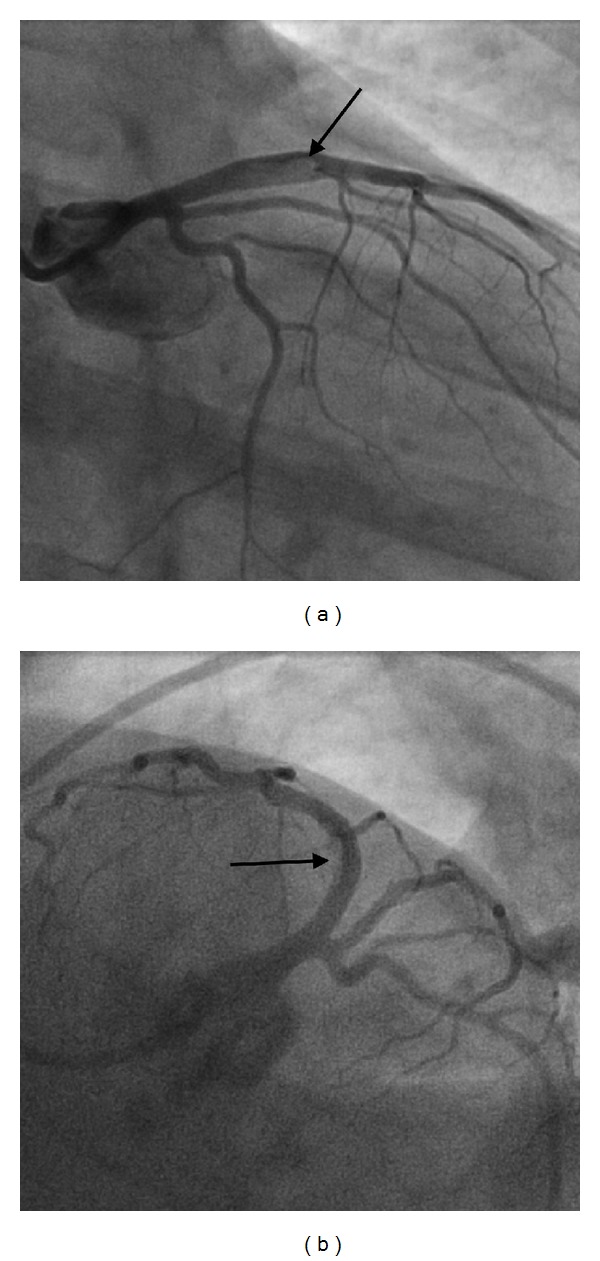
(a) 6 French diagnostic catheter in the right anterior oblique with caudal angulation. (b) In the left anterior oblique with caudal angulation (spider view). Arrow showing the presence of thrombus in the proximal left anterior descending artery.

**Figure 2:**
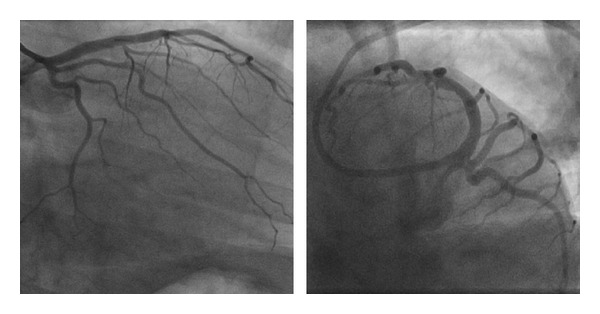
Complete resolution of thrombus in the same projection six weeks later.

A 45-year-old smoking male carpenter was referred to our center complaining of central chest heaviness radiating to his left arm. This was preceded by wasp stings 3 hours previously while gardening, resulting in the patient complaining of feeling weak, clammy, wheezy, and developing angioedema of the lips. His general practitioner treated him with intramuscular adrenaline, along with oral steroids and antihistamine thirty minutes following the initial wasp stings. Two hours after envenoment, he started complaining of chest discomfort. His electrocardiograph on presentation revealed sinus rhythm with T-wave inversion anteriorly. His troponin I levels were elevated at 0.5 mmol/L. Following episodes of ongoing rest pain, emergent cardiac catheterisation was performed. This revealed the presence of filling defect in the proximal left anterior descending artery consistent with a large clot burden (Figure 1). The rest of his coronaries appeared normal. Glycoprotein IIb/IIIa inhibitor was administered along with 5000 units of unfractionated heparin. Follow on percutaneous intervention (PCI) was performed utilising a rheolytic thrombectomy device (AngioJet, Possis Medical Europe), with significant clot extraction. Due to the nature of his presentation with anaphylaxis, we elected not to stent the thrombotic segment. Residual thrombus after thrombectomy was treated with therapeutic enoxaparin 1 mg/kg for the next five days. Repeat angiogram with intravascular ultrasound performed six weeks later revealed complete resolution of thrombus and normal coronary arteries (Figure 2). Serum wasp venom measured at six months confirmed wasp venom allergic hypersensitivity. He remained symptom-free at one-year followup, adhering to strict wasp sting avoidance strategy. 

## 3. Discussion

Common reactions following wasp (hymenoptera) stings include local envenoment site reaction that is self-limited and will resolve within hours. Anaphylaxis could occur in susceptible individuals and may manifest with limited cutaneous findings or in more extreme cases, systemic symptoms involving the gastrointestinal tract, genitourinary tract, or the nervous system (sense of impending doom, lightheadedness, and dizziness). Cardiopulmonary system involvement may progress rapidly to life-threatening cardiopulmonary collapse and manifest initially as breathing difficulties, bronchospasm, hypotension, and arrhythmia [1]. Myocardial infarction as a result of wasp stings is a rare manifestation of acute coronary syndromes with less than twenty documented reports in the literature [2]. The pathophysiological determinant seems to be related to the chemical composition of the venom made up by vasoactive and thrombogenic substances able to create vasospasm and coronary thrombosis as well as bioactive allergenic venom proteins such as phospholipase A1, hyaluronidases, and acid phosphatases [3]. The vasoactive mediator, histamine, can activate platelets and potentiates the aggregatory response of other agonists including adrenaline, 5-hydroxytryptamine, and thrombin. Histamine also induces proinflammatory cytokine production from endothelial cells [4]. Platelet aggregation and vasoconstriction is further induced by serotonin and thromboxanes and promotes the prothrombotic process [5, 6]. Increased platelet aggregations are seen in vivo when platelets are exposed to isolated wasp phospholipase A1 at nanomolar concentrations [7]. In our patient, exogenous epinephrine administration may have accentuated thrombus formation as it has been shown to promote platelet aggregation by increasing platelet production of thromboxane B2 [8, 9], heightening the sensitivity of platelets to ADP and promoting the thrombin-induced binding of platelets to fibrinogen [10]. Several reports of this condition identified patients as either at risk for endothelial dysfunction or else have coronary lesions de novo [11]. Rather than an isolated process resulting in mast cell degranulation and coronary vasospasm, it is the combination of all these factors in individuals with impaired endothelial function that may trigger an acute coronary syndrome. Our case depicts a rare phenomenon, whereby the resultant instantaneous thrombus formation was treated successfully utilising a glycoprotein IIb/IIIa inhibitor infusion in combination with low-molecular-weight heparin, negating the need for coronary stenting which in itself may promote localized hypersensitivity vasculitis contributing to late stent thrombosis [12] in a patient who may require future epinephrine therapy.

## 4. Conclusion

Acute coronary syndromes may occur in settings outside of plaque rupture. Awareness by general practitioner, paramedics, emergency physicians, and cardiologist is crucial to diagnose and treat this potential fatal scenario. Interventionist should remain cognizant to the various causes and treat each lesion taking into account the natural history, medication compliance, and consequences to coronary therapy.
